# Tracking the shifting landscape of SARS-CoV-2 variants in Lebanon among healthcare workers and hospitalized patients

**DOI:** 10.1128/spectrum.02254-25

**Published:** 2026-07-02

**Authors:** Habib Alkalamouni, Mirna Bou Hamdan, Farouk Abou Hassan, Fatima Dakroub, Lama Hamadeh, Alissar Rady, Moubadda Assi, Nada Ghosn, Rami Mahfouz, Carine Sakr, Nada Kara Zahreddine, Hassan Zaraket, Ghassan Dbaibo, Nada M. Melhem, Ghassan M. Matar

**Affiliations:** 1 Department of Experimental Pathology, Immunology and Microbiology, Faculty of Medicine, American University of Beirut 11238, Beirut, Lebanon; 2 Center for Infectious Diseases Research, Faculty of Medicine, American University of Beirut 11238, Beirut, Lebanon; 3 WHO Collaborating Center for Reference and Research on Bacterial Pathogens, Faculty of Medicine, American University of Beirut 11238, Beirut, Lebanon; 4 Medical Laboratory Sciences Program, Division of Health Professions, Faculty of Health Sciences, American University of Beirut 11238, Beirut, Lebanon; 5 Department of Pathology and Laboratory Medicine, Faculty of Medicine, American University of Beirut 11238, Beirut, Lebanon; 6 World Health Organization—Country Office—Beirut 66983, Beirut, Lebanon; 7 Lebanon Ministry of Public Health 485617, Beirut, Lebanon; 8 Department of Family Medicine, American University of Beirut 11238, Beirut, Lebanon; 9 Infection Control and Prevention Program, Faculty of Medicine, American University of Beirut 11238, Beirut, Lebanon; 10 Department of Pediatrics and Adolescent Medicine, Faculty of Medicine, American University of Beirut 11238, Beirut, Lebanon; Public Health Agency of Canada, Winnipeg, Manitoba, Canada

**Keywords:** SARS-CoV-2, genomic surveillance, healthcare workers, hospitalized patients, Omicron subvariants, Lebanon

## Abstract

Genomic surveillance of SARS-CoV-2 is critical for tracking viral evolution and informing public health responses. This study characterized variants circulating among healthcare workers (HCWs) and hospitalized patients in Lebanon between January 2022 and September 2024. A total of 530 SARS-CoV-2-positive nasopharyngeal swabs were collected from five Lebanese governorates and subjected to whole-genome sequencing. Correlations between variant circulation and a number of demographic and clinical variables were assessed. Most HCWs were female (64%), young adults (20–30 years, 39%), and had no comorbidities (97%). In contrast, hospitalized patients were mostly older adults (>60 years, 55.6%) with underlying conditions (77%). Early 2022 was marked by BA.1- and BA.2-like Omicron variants, followed by the predominance of BA.5-like lineages. In 2023, recombinant XBB sublineages became widespread. By 2024, these were largely replaced by next-generation variants, including JN.1 and KP.3.1.1. Despite differences in demographics and exposure risk, both groups showed parallel variant evolution. These findings reflect global and regional patterns and highlight the dynamic nature of SARS-CoV-2 circulation in Lebanon.

**IMPORTANCE:**

This study provides a comprehensive snapshot of SARS-CoV-2 variant evolution in Lebanon between 2022 and 2024, focusing on healthcare workers and hospitalized patients. By combining genomic and clinical data, it reveals how successive Omicron subvariants emerged and spread within key population groups. The detection of diverse and evolving lineages, including XBB recombinants and next-generation variants such as JN.1, underscores the ongoing antigenic drift of SARS-CoV-2. These insights reinforce the value of continued genomic surveillance for pandemic preparedness, especially in regions where data remain limited. Understanding local variant dynamics can guide targeted vaccination strategies and health policy decisions.

## INTRODUCTION

SARS-CoV-2, the causative agent of novel coronavirus disease 2019 (COVID-19), is known for its rapidly evolving variants, leading to reinfections and breakthrough infections among vaccinated and non-vaccinated individuals (1, 2). The original SARS-CoV-2 strain, Wuhan 19 (WA1/2020), gave rise to several subsequent variants categorized into variants under monitoring (VUM), variants of interest (VOI), and variants of concern (VOC) (3). This categorization was based on the Delphi consensus method used by the World Health Organization (WHO) Technical Advisory Group on Virus Evolution (TAG-VE) to monitor and classify the emerging SARS-CoV-2 variants (4–6). Lebanon followed the global trends of circulating SARS-CoV-2 variants, leading to five waves of COVID-19 (7). The first wave (December 2020 to February 2021) coincided with the detection of the first SARS-CoV-2 VOC, alpha (B.1.1.7). The second (February to July 2021) and third waves (July to October 2021) were predominated by the Delta VOC, a highly transmissible variant associated with severe disease. The Omicron variant, characterized by even higher transmissibility and severity, led the fourth (December 2021 to January 2022) and the fifth waves (June to September 2022) (8–11).

Since its emergence, the Omicron variant has become the predominantly circulating SARS-CoV-2 variant worldwide (12). This variant has been rapidly evolving into new subvariants, including BA.2.75, BA.4.6, BF.7, BA.5, BA.2.3.20, BA.2.75.2, BQ.1.1, and XBB, a recombinant of BJ.1 and BM.1.1.1 along with different sublineages; each categorized by the WHO into VOI or VUM (13–15). Among these, XBB was predominantly circulating globally in 2023 due to its high transmissibility and immune evasion; the former was used in the most recent version of the SARS-CoV-2 vaccines (16). In 2024, JN.1, SLip, FLiRT, KP.2, and KP.3.1.1, descendant lineages of BA.2.86, took over and became the most prevalent variants globally characterized by increased immune evasion relative to other variants circulating during the same period (17, 18). The continuous evolution of SARS-CoV-2 in 2024 has resulted in the emergence of various variants, including the currently circulating XEC and NB.1.8.1 lineages, underscoring the importance of continuous genomic surveillance and timely response to monitor emerging variants (19, 20).

It is well established that a large number of Omicron substitutions occur in the spike (S) protein, a critical site for entry to host cells and the primary target for neutralizing antibodies, in addition to several mutations in other regions (21, 22). These mutations (spike or non-spike) are known to impact pathogenesis as well as immune evasion (12, 23, 24). Notably, Omicron subvariants, including XBB, XBB.1, BQ.1, BQ.1.1, SLip, FLiRT, and KP.2, have demonstrated markedly reduced affinity for neutralizing antibodies derived from the sera of individuals previously infected with SARS-CoV-2 or those who received booster doses of bivalent (WA1/BA.5) mRNA vaccines (17, 25). While COVID-19 vaccines have effectively reduced morbidity, mortality, and hospitalizations associated with Omicron, mutations in the spike protein receptor-binding domain have enhanced the virus’s immune escape, contributing to increased breakthrough infections and reinfections (26, 27). These mutations, along with the potential emergence of severe variants and immune escape, raise serious concerns about the long-term effectiveness of current mRNA vaccines (28). This underscores the urgent need for updated vaccine formulations specifically targeting the evolving mutations in newly emerging subvariants (17, 25, 29, 30).

The continuous evolution of the virus, coupled with the occurrence of breakthrough infections among healthcare workers (HCWs) and hospitalized patients (31–48), highlights the critical need for genomic surveillance to track and monitor variant emergence to inform public health strategies (49–51). To address this need, we initiated in 2021 a national SARS-CoV-2 genomic surveillance program to monitor the circulation of SARS-CoV-2 among HCWs in Lebanon. Genomic sequencing of samples collected from HCWs between December 2021 and January 2022 showed similar trends to those reported globally during the same period of time (49).

In this study, we build on that effort by characterizing the ongoing evolution and distribution of SARS-CoV-2 variants among HCWs and hospitalized patients in Lebanon between January 2022 and September 2024.

## MATERIALS AND METHODS

### Study design, population, and data collection

This study was part of a national surveillance program conducted in collaboration with the Epidemiological Surveillance Unit at the Lebanese Ministry of Public Health and the WHO to monitor trends and evolution of SARS-CoV-2 among hospitalized patients and HCWs. We established a laboratory network across a number of governorates. The network was used to collect nasopharyngeal samples from any reported SARS-CoV-2-positive hospitalized patient and HCW in order to monitor the national evolution of SARS-CoV-2. A total of 530 nasopharyngeal swabs were collected from COVID-19-positive hospitalized patients (*n* = 126) and HCWs (*n* = 404) between January 2022 and September 2024 from 16 Lebanese healthcare centers within this network. The laboratory network included healthcare centers distributed as follows: Beirut (AUB Medical Center: *n* = 199, Saint George Hospital University Medical Center: *n* = 41, Makassed General Hospital: *n* = 30, and Rafic Hariri Medical Center: *n* = 2), Mount Lebanon (Kesserwan Medical Center: *n* = 2, Ain W Zein Hospital: *n* = 57, Mount Lebanon Hospital: *n* = 23, Hôpital Notre Dame des Secours: *n* = 15, and Belle Vue Hospital: *n* = 19), South Lebanon (Hammoud Hospital: *n* = 69), Bekaa (Bekaa Hospital: *n* = 14, Tel Chiha Hospital: *n* = 1, and Elias Hrawi Governmental Hospital: *n* = 9), and North Lebanon (New Mazloum Hospital: *n* = 9, Nini Hospital: *n* = 37, and North Medical Center: *n* = 3). Samples with cycle threshold (Ct) values below 25 were included. All collected samples were stored at −80°C until processing. When available, demographic and clinical data were retrieved from medical records, including the date of positive PCR test, age, gender, vaccination status, number of received vaccine doses, occupation, and comorbidities.

### RNA extraction and genomic sequencing

Viral RNA was extracted from nasopharyngeal samples using QIAamp Viral RNA mini-Kit (QIAGEN, Hilden, Germany, Cat. 52906) according to the manufacturer’s instructions, with the addition of 1 min incubation at room temperature prior to elution and an adjustment of the elution volume to 30 µL. The concentration and quality of RNA samples were measured and checked with the Denovix Blue DS-11 Spectrophotometer.

Aliquots of RNA extracts (*n* = 530, Ct ≤ 25) were used to perform SARS-CoV-2 genomic sequencing. Sequencing results were available for a total of 357 samples (*n* = 231 HCW samples; *n* = 126 hospitalized samples); the remaining samples either failed the quality control check or did not yield enough reads for analysis. The Illumina (*n* = 187) and Oxford Nanopore (*n* = 343) sequencing platforms were used as described below.

#### Genomic sequencing using Illumina

A total of 187 RNA extracts were processed for genomic sequencing using COVIDseq Assay Index I and Miseq Reagent kit (Illumina, Document 1000000126053, v07 guidelines) as previously described (52). Briefly, random hexamers were annealed to the extracted RNA, followed by cDNA synthesis and amplification using the ARTIC v4 primer pools, generating 400 bp amplicons in the process. These amplicons were then fragmented and tagged with adapter sequences through tagmentation, cleaned, and further amplified with indexing sequencing i7 and i5 to facilitate cluster generation. The amplicons were pooled, purified, and quantified using the Qubit 1× dsDNA High Sensitivity (HS) assay using the Qubit 4 fluorometer (Invitrogen). The library was then diluted to 4 nM and loaded onto a MiSeq sequencing device at a concentration of 6 pM following denaturation using the MiSeq Reagent Kit v2 (Protocol A). The resulting FASTQ files were analyzed on the Illumina BaseSpace Sequence Hub using the DRAGEN Bio-IT Platform to identify viral variants. This process ensured a 30× genome coverage for precise variant detection and analysis of variant distribution (52).

#### Genomic sequencing using Oxford Nanopore

A total of 343 RNA extracts were sequenced using the Oxford Nanopore MK1C device as previously described (53). Briefly, 8 µL of extracted RNA was used for cDNA synthesis using the Midnight RT-PCR Expansion Kit with the V3 primer scheme, designed to produce 1,200 bp amplicons. Following amplification using two distinct primer pools, the resulting amplicons were pooled, barcoded using the Rapid Barcoding Kit V10 or V14, and then purified with AMPure XP Beads. The DNA library was quantified using the Qubit 1× dsDNA HS assay. Depending on the batch size, 200 ng or 800 ng of the library was loaded onto Flongle or standard SpotON flow cells, respectively. The flow cells were primed and loaded with a mix of sequencing buffer and library beads without introducing bubbles. Sequencing parameters were adjusted as follows: barcoding enabled, basecalling enabled, and overriding both the minimum barcoding score and minimum mid-read barcoding score to values of 60 and 50, respectively. The FASTQ files generated were analyzed using the epi2me-labs/wf-artic pipeline, tailored for handling Oxford Nanopore data with the following adjustments: the primer scheme was set to “Midnight-ONT/V3” to align with the specific primers used in the sequencing, and the basecaller was selected based on compatibility with the particular barcoding chemistry kit employed. A minimum coverage of 20× was necessary for variant calling.

### Phylogenetic tree construction

For the construction of the phylogenetic tree of SARS-CoV-2 sequences, a comprehensive data set from both Illumina and Oxford Nanopore platforms was compiled by combining individual FASTA files into a single file. This combined data set was aligned using the MAFFT alignment tool with the command mafft --auto --large to accommodate the large data set. The alignment output was exported into FASTA format, and subsequently, the PhyML software was employed for phylogenetic analysis using the GTR model of nucleotide substitution with a bootstrap analysis of 100 replicates to assess the reliability of the inferred phylogenetic tree (54). The resulting tree was then visualized and refined using the Interactive Tree Of Life (iTOL) web tool, allowing for the graphical representation and annotation of the phylogenetic relationships among the viral sequences.

### Statistical analysis

Data analysis was performed using STATA SE 13.0. Descriptive statistics, including frequencies, percentages, and means, were calculated. SARS-CoV-2 variants were categorized into four groups according to periods of variant predominance, with classifications performed separately for HCWs and hospitalized patients, as well as for a pooled cohort combining both populations. We performed univariate logistic regression analyses to assess factors associated with infection by predominant variants (dependent variable). Univariate logistic regression analyses were conducted for HCWs, hospitalized patients, and the combined cohort. In each model, infection with a predominant Omicron subvariant was specified as the outcome, with other non-predominant Omicron variants serving as the reference category. Depending on the data available for each of HCWs and hospitalized patients, each predictor was assessed individually and included age, sex, year of sample collection, vaccination status, HCW role, number of vaccine doses received, time elapsed between the most recent vaccine dose and infection, presence of comorbidities, and site of sample collection (Beirut governorate vs other governorates). Observations with missing variant data were excluded from all analyses. A *P* value < 0.05 was considered significant. Figures were generated using GraphPad Prism 9.3.1.

## RESULTS

### Demographic and clinical data of study participants

**Fig 1:**
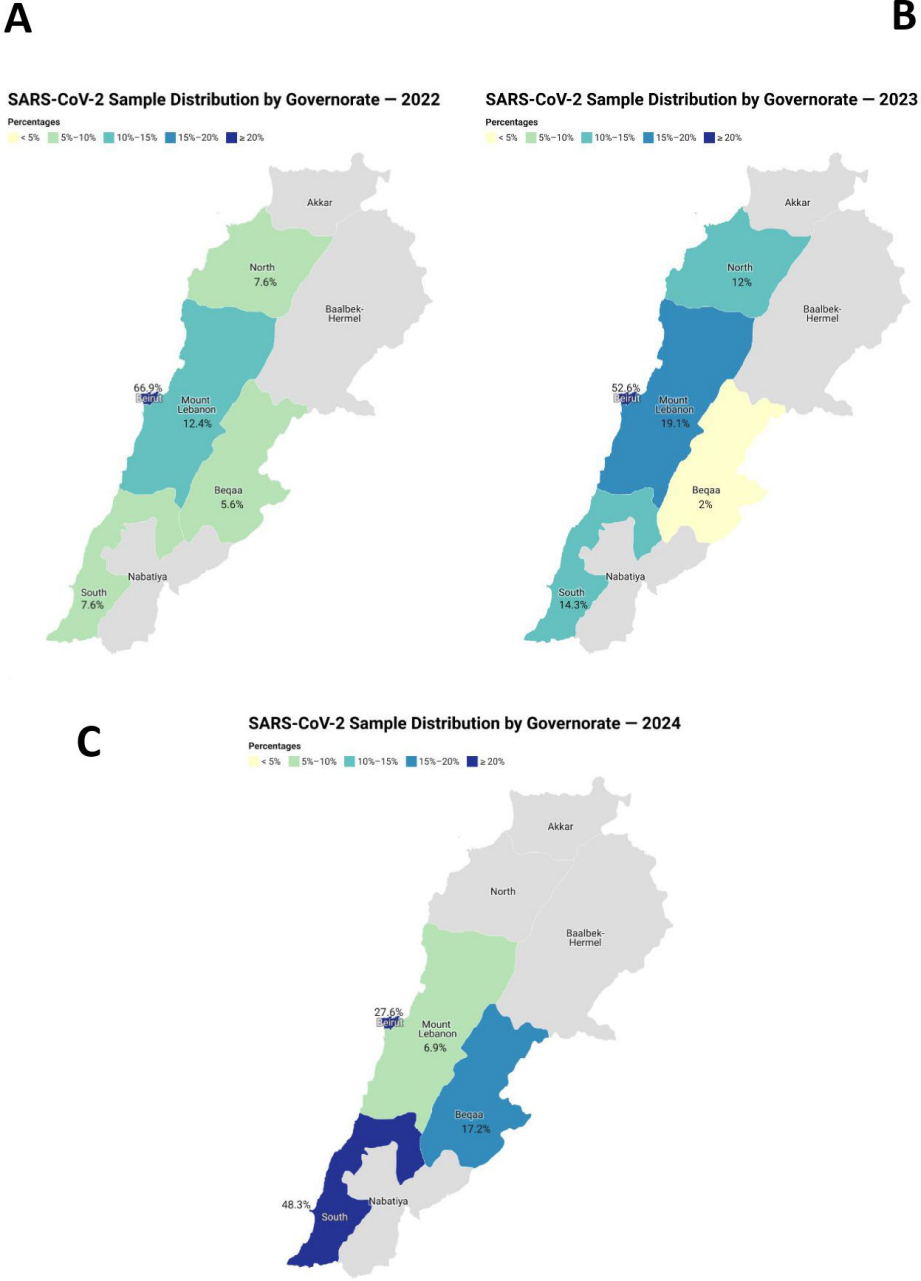
Geographic distribution of SARS-CoV-2 samples across Lebanese governorates (2022–2024). Proportional distribution of SARS-CoV-2 samples collected from Lebanese governorates per year: (**A**) 2022; (**B**) 2023; (**C**) 2024. The color coding depicted (where applicable) is linked to percentages of samples by governorate. Gray shading indicates governorates from which no samples were collected during the corresponding year. The maps were constructed using the online tool found on the website simplemaps.com.

Between January 2022 and September 2024, 530 nasopharyngeal swabs were collected from PCR-positive HCWs (*n* = 404) and hospitalized patients (*n* = 126) across five Lebanese governorates (Fig. 1). Demographic and clinical data were available for all hospitalized patients and 67% of HCWs (Table 1). HCW samples were collected in 2022 and 2023; no PCR-positive HCWs were collected in 2024 (Table 1).

**TABLE 1:** Demographic, testing, and vaccination characteristics of study participants^*a*^

	*N*	%
HCWs (*n* = 404)		
Gender (*n* = 270)		
Male	97	35.92
Female	173	64.08
Age (*n* = 113)		
20–30 years	44	38.94
31–40 years	23	20.35
41–50 years	23	20.35
51–60 years	13	11.50
>60 years	10	8.85
Position (*n* = 244)		
Physician	67	27.46
Nurse	127	52.04
Others	50	20.50
PCR date (*n* = 404)		
January to April 2022	125	30.94
May to September 2022	67	16.59
October to December 2022	19	4.7
January to April 2023	117	28.96
May to September 2023	76	18.82
COVID-19 vaccination (*n* = 232)		
Yes	229	98.70
No	3	1.30
Vaccine doses (*n* = 122)		
One dose	2	1.64
Two doses	28	22.95
Three doses	86	70.50
Four doses	6	4.91
Duration between last vaccine dose and infection (*n* = 63)		
<1 year	19	30.16
1–2 years	42	66.67
2–3 years	2	3.17
Hospitalized patients (*n* = 126)		
Gender (*n* = 126)		
Male	65	51.59
Female	61	48.41
Age (*n* = 126)		
0–20 years	29	23.02
20–30 years	3	2.38
31–40 years	3	2.38
41–50 years	6	4.76
51–60 years	15	11.90
>60 years	70	55.56
PCR date (*n* = 126)		
January to April 2022	35	27.78
May to September 2022	0	0
October to December 2022	4	3.17
January to April 2023	26	20.63
May to September 2023	24	19.05
October to December 2023	8	6.35
January to April 2024	10	7.94
May to September 2024	19	15.08
COVID-19 vaccine status (*n* = 36)		
Yes	19	52.78
No	17	47.22
Vaccine doses (*n* = 19)		
One dose	0	0
Two doses	8	42.10
Three doses	9	47.37
Four doses	2	10.53

^*a*^ Others included lab technicians, pharmacists, OR technicians, ER staff, physiotherapists, radiographers, clerks, and tellers.

The majority of HCWs were female (64%), aged 20–30 years (39%), and working in Beirut (58.4%), with nurses comprising 52% of the cohort. Most HCWs (97%) reported no comorbidities. Infections were more frequent in 2022 than in 2023. Nearly all HCWs (98.7%) were vaccinated with the Pfizer-BioNTech COVID-19 vaccine prior to July 2022. Among vaccinated HCWs, 70.5% received three doses, 23% received two doses, 5% received four doses, and 1.5% received a single dose. The majority of infections occurred more than 1 year after the last vaccine dose.

In contrast, hospitalized patients were predominantly males (51.6%) and over 60 years of age (55.5%). Most had comorbid conditions (77%), particularly cardiovascular disease (57.6%) and diabetes mellitus (28.8%) (data not shown). The majority of hospitalizations occurred in 2023. Vaccination data were available for 36 patients; of these, 52.8% had received the vaccine, either two (42%) or three (47%) doses. Due to missing vaccine dates, the time since vaccination could not be determined.

### Variant distribution and evolution

Samples were collected from healthcare centers across five governorates in 2022 and 2023 (Fig. 1A and B). The majority of these samples were collected from Beirut (66.9% in 2022; 52.6% in 2023), followed by Mount Lebanon (12.4% in 2022; 19.1% in 2023) and South Lebanon (7.6% in 2022; 14.3% in 2023). In 2024, sampling was limited to hospitalized patients from four governorates (Beirut, Mount Lebanon, Bekaa, and South Lebanon), with the highest proportion obtained from South Lebanon (Fig. 1C). Across all years, most samples were collected in Beirut (*n* = 307), particularly during early 2022.

**Fig 2:**
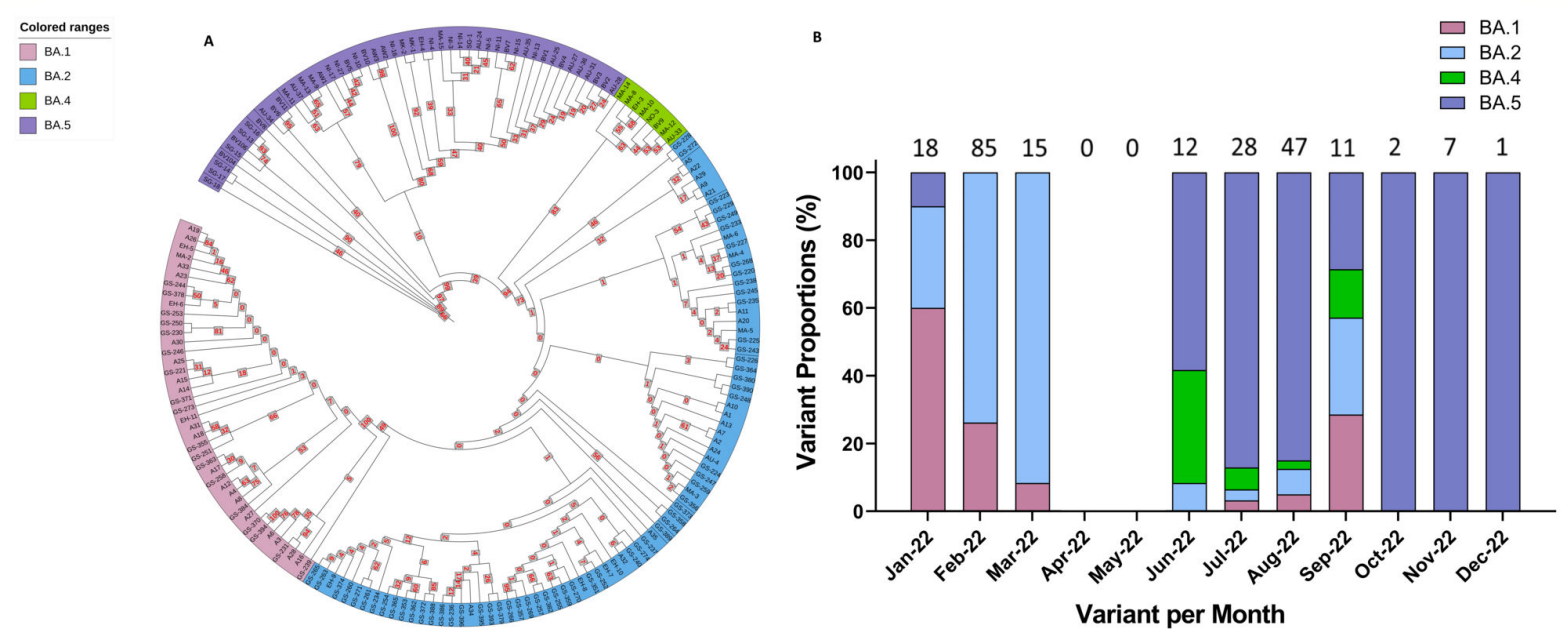
SARS-CoV-2 phylogeny and temporal variant distribution among healthcare workers in 2022. (**A**) Maximum-likelihood phylogenetic tree of SARS-CoV-2 genomes detected among HCWs in 2022. Tips are colored by Omicron lineages (BA.1, BA.2, BA.4, and BA.5). Bootstrap support values (100 replicates) are shown at internal nodes. (**B**) Proportions of Omicron lineages detected among HCWs per month in 2022. Numbers above bars indicate the total number of sequences analyzed per month. Blank months represent periods during which no samples were available for sequencing for the corresponding cohort.

**Fig 3:**
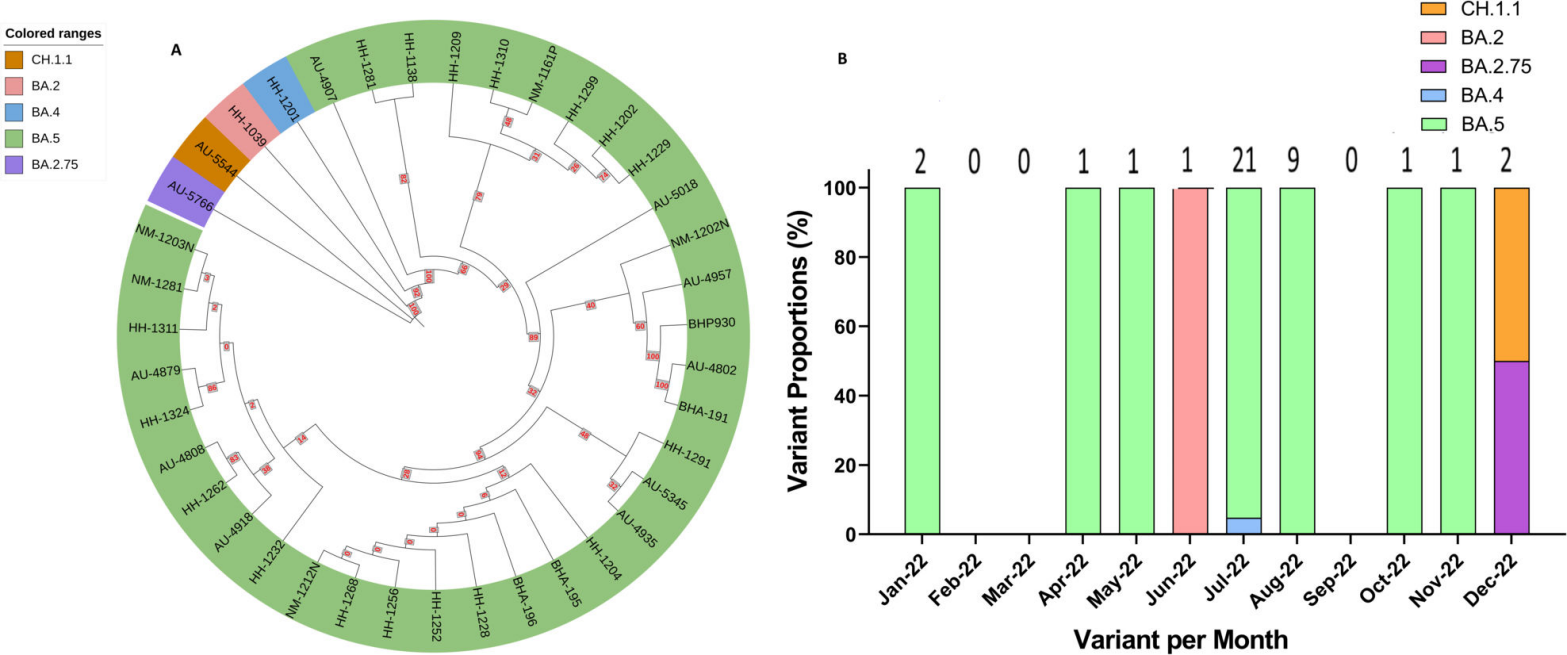
SARS-CoV-2 phylogeny and temporal variant distribution among hospitalized patients in 2022. (**A**) Maximum-likelihood phylogenetic tree of SARS-CoV-2 genomes detected among hospitalized patients in 2022. Tips are colored by Omicron lineages (BA.2, BA.4, BA.5, BA.2.75, and CH.1.1). Bootstrap support values (100 replicates) are shown at internal nodes. (**B**) Proportions of Omicron lineages detected among hospitalized patients per month in 2022. Numbers above bars indicate the total number of sequences analyzed per month.

**Fig 4:**
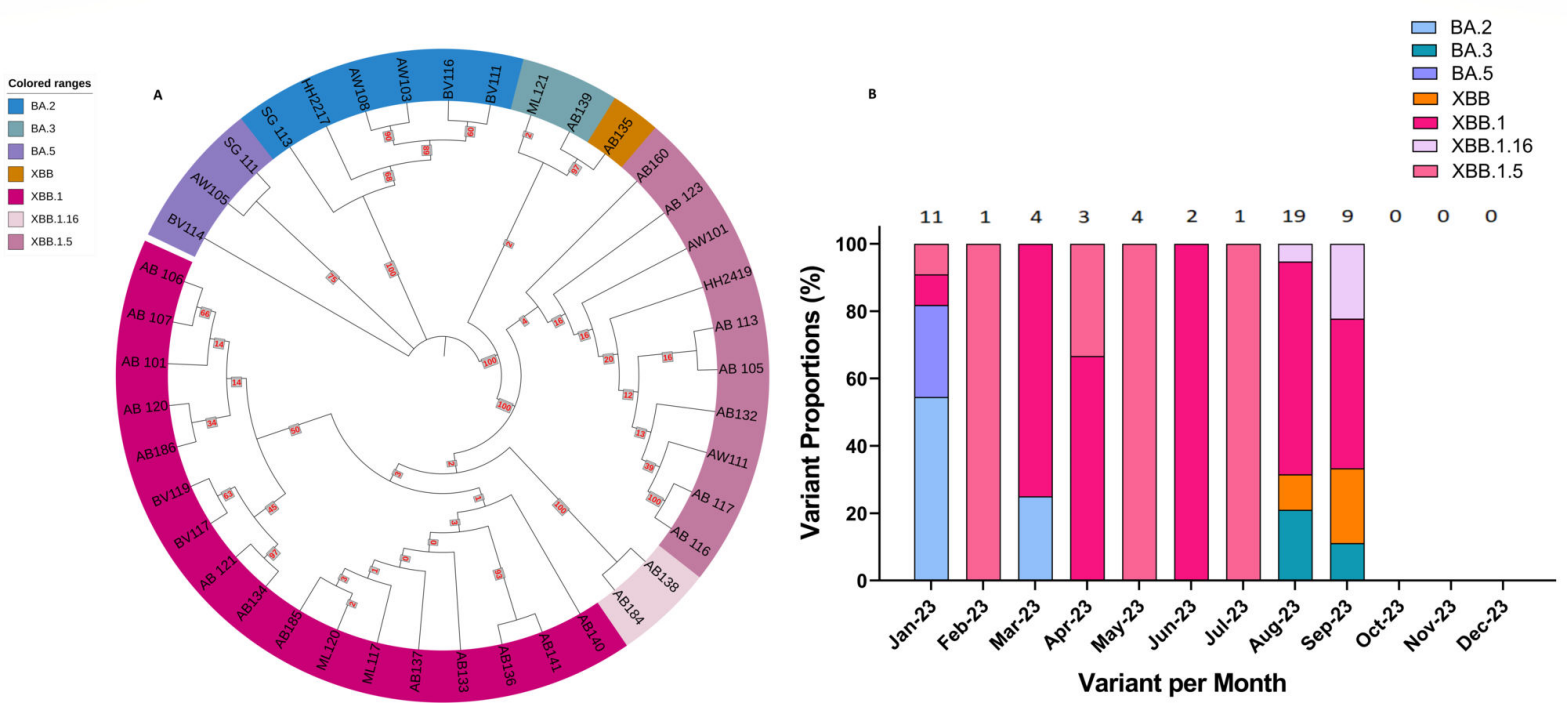
SARS-CoV-2 phylogeny and temporal variant distribution among healthcare workers in 2023. (**A**) Maximum-likelihood phylogenetic tree of SARS-CoV-2 genomes detected among HCWs in 2023. Tips are colored by Omicron and recombinant lineages (BA.2, BA.3-, BA.5-, XBB-, and XBB-derived lineages). Bootstrap support values (100 replicates) are shown at internal nodes. (**B**) Proportions of Omicron lineages detected among HCWs per month in 2023. Numbers above bars indicate the total number of sequences analyzed per month.

**Fig 5:**
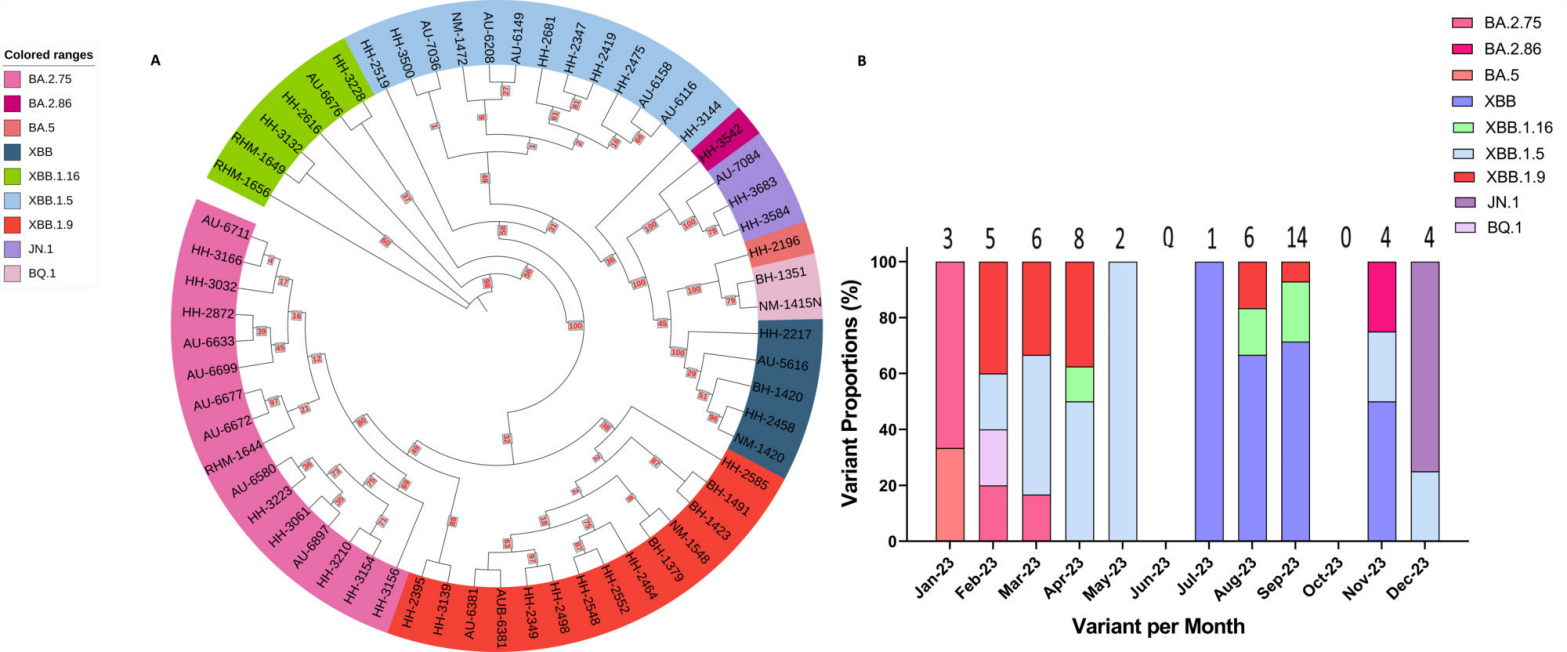
SARS-CoV-2 phylogeny and temporal variant distribution among hospitalized patients in 2023. (**A**) Maximum-likelihood phylogenetic tree of SARS-CoV-2 genomes detected among hospitalized patients in 2023. Tips are colored by Omicron and recombinant lineages (BA.2.75-, BA.2.86-, BA.5-, XBB-, XBB-derived lineages, JN.1, and BQ.1). Bootstrap support values (100 replicates) are shown at internal nodes. (**B**) Proportions of Omicron lineages detected among hospitalized patients per month in 2023. Numbers above bars indicate the total number of sequences analyzed per month.

**Fig 6:**
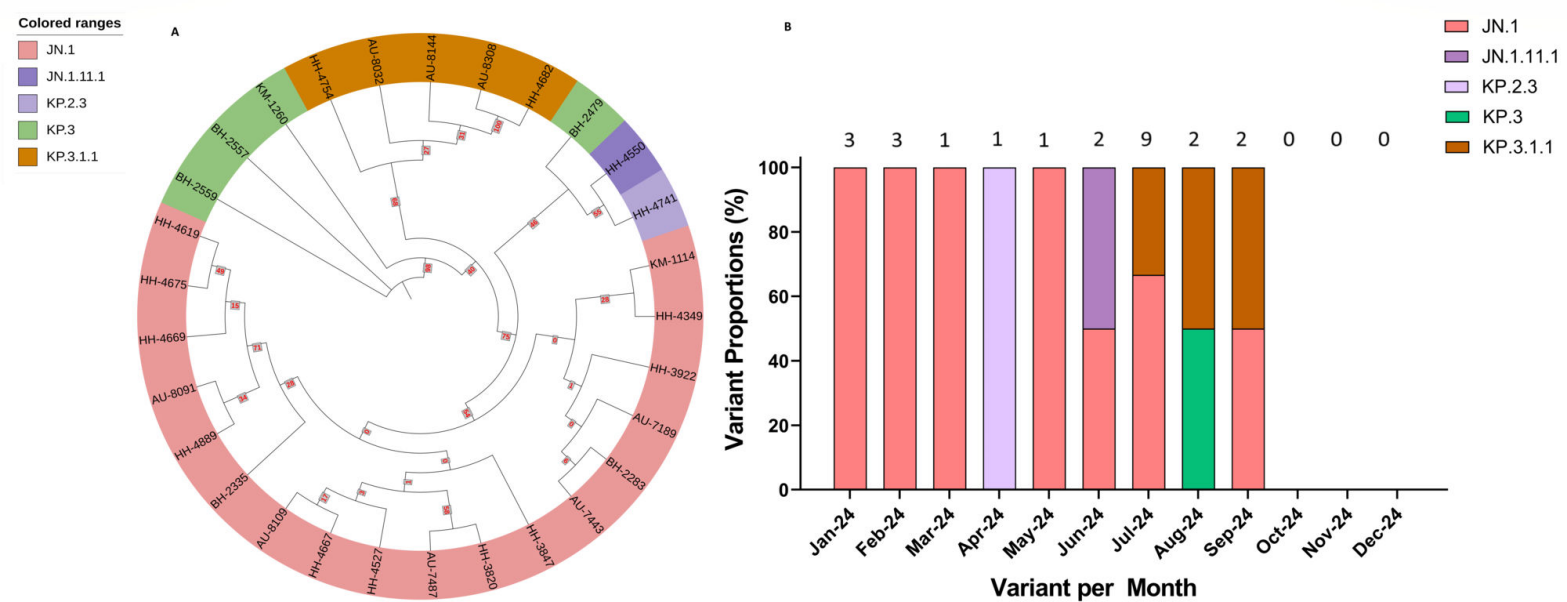
SARS-CoV-2 phylogeny and temporal variant distribution among hospitalized patients in 2024. (**A**) Maximum-likelihood phylogenetic tree of SARS-CoV-2 genomes detected among hospitalized patients in 2024. Tips are colored by Omicron lineages and descendants (JN.1, JN.1.11.1, KP.2.3, KP.3, and KP.3.1.1). Bootstrap support values (100 replicates) are shown at internal nodes. (**B**) Proportions of Omicron lineages detected among hospitalized patients per month in 2024. Numbers above bars indicate the total number of sequences analyzed per month.

Phylogenetic analysis of SARS-CoV-2 sequences from HCWs and hospitalized patients in Lebanon reveals a dynamic and parallel pattern of viral evolution, marked by successive waves of Omicron sublineages and increasing genetic diversity (Fig. 2 to 6). In early 2022, infections among HCWs were primarily associated with closely related BA.1- and BA.2-like variants, forming tight clusters with minimal divergence, consistent with restricted viral diversity and limited transmission chains within healthcare settings (Fig. 2A) along with temporal dominance of these lineages during the first half of the year (Fig. 2B). In contrast, hospitalized patients were already transitioning to BA.5-like variants—particularly BA.5.2, BA.5.1, and BA.5.2.1—which dominated by mid-2022 and formed compact clades with strong bootstrap support (Fig. 3A). Additional divergent variants, including BE.1.1-, BF.5-, and CH.1.1-like, were also observed among hospitalized individuals, possibly reflecting separate community-based introductions, variant-specific clinical associations, or differences in sampling. Temporal bar plots support these observations, showing an earlier and more rapid shift to BA.5 in hospitalized patients (Fig. 3B). As 2022 progressed, both groups converged toward BA.5 dominance. This transition occurred earlier in hospitalized patients, while HCWs showed a more gradual and heterogeneous mid-year transition, characterized by the replacement of BA.2 by BA.5 and a brief, transient circulation of BA.4 prior to convergence.

In 2023, the phylogenies became increasingly complex, driven by the emergence of recombinant Omicron sublineages. Among HCWs, a diverse lineage profile was observed, including BA.2- and BA.5-derived lineages, as well as multiple XBB-derived recombinants (Fig. 4A). Hospitalized patients also exhibited a broad range of lineages—dominated by XBB-derived sublineages and including several well-supported, divergent branches (Fig. 5A). XBB-derived lineages became particularly dominant in the second half of the year in both groups (Fig. 4B and 5B). Both groups experienced overlapping but distinct variant distributions throughout 2023, with XBB-derived sublineages gradually replacing earlier Omicron lineages. These findings underscore the parallel evolutionary trajectories of SARS-CoV-2 in both populations, suggesting overlapping exposures and potentially independent introductions of circulating variants into each group.

**Fig 7:**
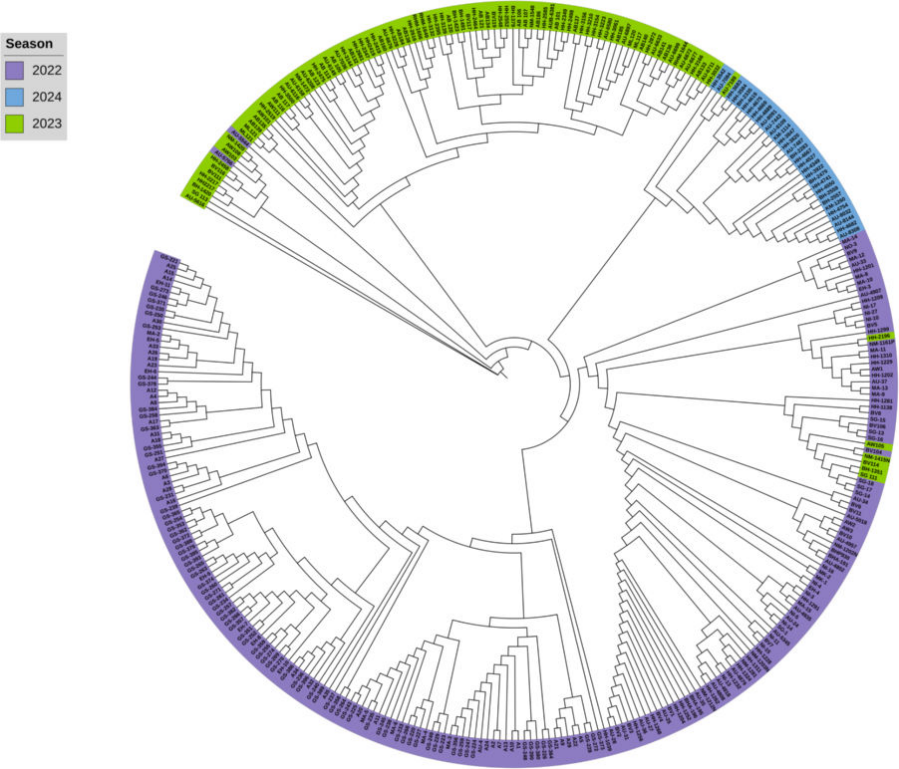
Phylogenetic overview of SARS-CoV-2 genomes from HCWs and hospitalized patients between 2022 and 2024. Maximum-likelihood phylogenetic tree of all SARS-CoV-2 genomes analyzed from HCWs and hospitalized patients in this study, colored by year of collection (2022, 2023, and 2024). The tree illustrates the temporal progression and diversification of circulating Omicron lineages across the 3-year surveillance period.

By 2024, hospitalized patient sequences showed marked divergence from earlier Omicron lineages. During the first few months, genomes were predominantly JN.1, while mid- and late-year samples revealed the appearance of KP.3 and KP.3.1.1, which formed distinct, distal branches, consistent with continued antigenic drift and diversification (Fig. 6A). These transitions are also reflected in Fig. 6B, where JN.1 was progressively replaced by KP-derived lineages. Longitudinal analysis across the 3-year period (Fig. 7) highlights sustained evolution, illustrating how successive variant waves contributed to the expanding genetic landscape of SARS-CoV-2 in hospitalized individuals.

### Variant correlation with demographic and clinical characteristics

Among HCWs, predominant variants included Omicron BA.1, BA.2, and BA.5 variants (Table 2). Our results showed that HCWs from governorates outside Beirut had significantly lower odds of BA.1 (β = −1.934, *P* = 0.001) and BA.2 (β = −4.959, *P* < 0.001) infection compared to those from Beirut. In contrast to BA.1 and BA.2, individuals from other governorates had significantly higher odds of BA.5 infection compared to Beirut (β = 0.814, *P* = 0.049). Compared with physicians, HCWs categorized as “Others” (including lab technicians, pharmacists, radiologists, emergency room staff, operating room staff, inhalation technicians, phlebotomists, hospital tellers, and clerks) had significantly reduced odds of BA.1, BA.2, and BA.5 infection.

**TABLE 2:** Relationship between predominant Omicron variants among HCWs and demographic and clinical characteristics^*a*^

Variant	Variables	Coefficient	Standard error	*P*-value^*c*^	95% Confidence interval
Omicron BA.1					
	Gender (*n* = 171)				
	Male	Ref			
	Female	−0.530	0.628	0.399	−1.762 to 0.701
	Age (*n* = 27)				
	≤60 years	Ref			
	>60 years	9.95e-10	6,911.327	1.000	−13545.95 to 13,545.95
	Site of sample collection (*n* = 230)				
	Beirut governorate	Ref			
	Other governorates	−1.934	0.605	**0.001**	−3.120 to 0.748
	Year of sample collection (*n* = 230)				
	2022	Ref			
	2023	−19.546	1,369.114	0.989	−2702.962 to 2,663.869
	HCWs position (*n* = 230)				
	Physicians	Ref			
	Nurses	−1.373	1.148	0.232	−3.624 to 0.878
	Physiotherapists	−2.780	1.437	0.060	−5.525 to 1.109
	Others^*b*^	−4.959	1.158	**0.000**	−7.230 to 2.687
	SARS-CoV-2 vaccination (*n* = 157)	2.75e-08	–^*d*^	–	–
Omicron BA.2					
	Gender (*n* = 171)				
	Male	Ref			
	Female	−0.480	0.582	0.410	−1.621 to 0.661
	Age (*n* = 27)				
	≤60 years	Ref			
	>60 years	9.95e-10	6,911.327	1.000	−13,545.95 to 13,545.95
	Site of sample collection (*n* = 230)				
	Beirut governorate	Ref			
	Other governorates	−1.455	0.415	**0.000**	−2.269 to −0.640
	Year of sample collection (*n* = 230)				
	2022	Ref			
	2023	−4.076	0.562	**0.000**	−5.178 to 2.963
	HCWs position (*n* = 230)				
	Physicians	Ref			
	Nurses	−1.003	1.124	0.372	−3.207 to 1.200
	Physiotherapists	−1.473	1.277	0.249	−3.977 to 1.030
	Others^*b*^	−4.250	1.070	**0.000**	−6.347 to −2.153
	SARS-CoV-2 vaccination (*n* = 157)	4.71e-08	3,980.57	1.000	−7,801.773 to 7,801.773
Omicron BA.5					
	Gender (*n* = 171)				
	Male	Ref			
	Female	−0.046	0.662	0.944	−1.344 to 1.251
	Age (*n* = 27)				
	≤60 years	Ref			
	>60 years	16.518	4,887.078	1.000	−9,561.917 to 9,594.954
	Site of sample collection (*n* = 230)				
	Beirut governorate	Ref			
	Other governorates	0.814	0.414	**0.049**	0.003–1.625
	Year of sample collection (*n* = 230)				
	2022	Ref			
	2023	−4.206	0.701	**0.000**	−5.581 to 2.832
	HCWs position (*n* = 230)				
	Physicians	Ref			
	Nurses	−0.855	1.176	0.467	−3.161 to 1.449
	Physiotherapists	−0.693	1.322	0.600	−3.285 to 1.899
	Others^*b*^	−2.772	1.097	**0.012**	−4.923 to −0.622
	SARS-CoV-2 vaccination (*n* = 157)	−17.287	3,151.908	0.996	−6,194.913 to 6,160.338
Other Omicron variants	(Base outcome)				

^*a*^ Univariate logistic regression. Vaccine doses variable and the date between the last vaccination dose and infection variable were omitted due to limited variability. The comorbidities variable was also omitted due to collinearity.

^*b*^ Others include lab technicians, pharmacists, radiologists, emergency room staff, operating room staff, inhalation technicians, phlebotomists, hospital tellers, and clerks.

^*c*^ Bold values indicate statistically significant relationships (*P* < 0.05).

^*d*^ ”–” indicates omitted/empty results in the univariate regression analysis.

Similar analyses were also conducted to evaluate the association between selected predictors and infection with Omicron variants (BA.5, JN.1, and XBB) among hospitalized patients, using other Omicron variants as the reference outcome (Table 3). Overall, demographic characteristics, including gender and age, were not significantly associated with any of the evaluated Omicron variants. Although older age (>60 years) showed a non-significant trend toward increased odds of infection with the JN.1 variant (β = 0.944, *P* = 0.132), this association did not reach statistical significance. The year of sample collection emerged as the only consistent predictor across models, although its direction of association varied by variant. Compared to 2022, samples collected in 2023 and 2024 were associated with significantly lower odds of infection with the BA.5 variants (2023: β = −3.620, *P* = 0.002; 2024: β = −2.772, *P* = 0.001). Conversely, the odds of infection with the XBB variants were significantly higher in 2023 (β = 3.652, *P* = 0.002), while no significant association was observed for 2024 due to substantial estimate imprecision. For the JN.1 variant, the year of sample collection was not significantly associated with infection, with extremely large standard errors and wide confidence intervals indicating model instability.

**TABLE 3:** Relationship between predominant Omicron variants among hospitalized patients and demographic and clinical characteristics^*a*^

Variant	Variables	Coefficient	Standard error	*P*-value^*b*^	95% Confidence interval
Omicron BA.5					
	Gender (*n* = 126)				
	Male	Ref			
	Female	0.367	0.546	0.501	−0.704 to 1.439
	Age (*n* = 126)				
	≤60 years	Ref			
	>60 years	0.405	0.543	0.456	−0.660 to 1.471
	Year of sample collection (*n* = 126)				
	2022	Ref			
	2023	−3.620	1.157	**0.002**	−5.889 to −1.350
	2024	−2.772	0.801	**0.001**	−4.343 to −1.201
	Site of sample collection (*n* = 126)				
	Beirut governorate	Ref			
	Other governorates	0.117	0.614	0.848	−1.086 to 1.321
	SARS-CoV-2 vaccination (*n* = 36)	16.625	2,353.274	0.994	−4,595.707 to 4,628.958
	SARS-CoV-2 vaccine doses (*n* = 36)				
	≤2 doses	Ref			
	≥3 doses	−17.070	5,880.237	0.998	−11,542.12 to 11,507.98
	Comorbid conditions (*n* = 126)	−0.058	0.583	0.920	−1.202 to 1.084
Omicron JN.1					
	Gender (*n* = 126)				
	Male	Ref			
	Female	0.185	0.609	0.761	−1.008 to 1.379
	Age (*n* = 126)				
	≤60 years	Ref			
	>60 years	0.944	0.626	0.132	−0.284 to 2.172
	Year of sample collection (*n* = 126)				
	2022	Ref			
	2023	15.835	1,326.24	0.990	−2,583.547 to 2,615.217
	2024	16.808	1,326.24	0.990	−2,582.574 to 2,616.19
	Site of sample collection (*n* = 126)				
	Beirut governorate	Ref			
	Other governorates	1.77e-15	0.677	1.000	−1.326 to 1.326
	SARS-CoV-2 vaccination (*n* = 36)	−0.200	0.775	0.796	−1.721 to 1.320
	SARS-CoV-2 vaccine doses (*n* = 36)				
	≤2 doses	Ref			
	≥3 doses	0.539	0.929	0.562	−1.281 to 2.360
	Comorbid conditions (*n* = 126)	−1.540	0.871	0.077	−3.248 to 0.167
Omicron XBB					
	Gender (*n* = 126)				
	Male	Ref			
	Female	0.454	0.524	0.386	−0.573 to 1.482
	Age (*n* = 126)				
	≤60 years	Ref			
	>60 years	0.356	0.520	0.493	−0.663 to 1.376
	Year of sample collection (*n* = 126)				
	2022	Ref			
	2023	3.652	1.154	**0.002**	1.390 to 5.914
	2024	−14.539	1,172.436	0.990	−2,312.471 to 2,283.393
	Site of sample collection (*n* = 126)				
	Beirut governorate	Ref			
	Other governorates	−0.254	0.572	0.656	−1.377 to 0.867
	SARS-CoV-2 vaccination (*n* = 36)	−16.777	2,539.03	0.995	−4,993.185 to 4,959.63
	SARS-CoV-2 vaccine doses (*n* = 36)				
	≤2 doses	Ref			
	≥3 doses	18.451	2,931.405	0.995	−5,726.998 to 5,763.9
	Comorbid conditions (*n* = 126)	−0.651	0.589	0.269	−1.807 to 0.504
Other Omicron variants	(Base outcome)				

^*a*^ Univariate logistic regression.

^*b*^ Bold values indicate statistically significant relationships (*P* < 0.05).

We performed similar analyses to assess the association between participant characteristics and infection with Omicron variants (BA.5, BA.2, and XBB), using other Omicron variants as the reference outcome (Table 4). Overall, participant type (HCWs vs hospitalized patients) was not significantly associated with infection for any of the evaluated variants (Table 4). Similarly, gender and age were not significantly associated with variant-specific infection across all models. For BA.5, the presence of comorbid conditions was associated with higher odds of infection (β = 1.317, *P* = 0.005). In addition, samples collected in 2023 and 2024 were less likely to be BA.5 compared with those from 2022 (β = −2.892 and −2.546, respectively; both *P* < 0.001). Individuals from governorates outside Beirut also had higher odds of BA.5 infection (β = 1.040, *P* < 0.001). Vaccination status and number of doses were not associated with BA.5. For BA.2, most variables were not associated with infection and showed imprecise estimates. However, receipt of ≥3 vaccine doses was associated with higher odds of BA.2 infection (β = 1.704, *P* = 0.033). Samples from 2023 had lower odds compared with 2022 (β = −2.547, *P* < 0.001), and residence outside Beirut was associated with reduced odds (β = −1.438, *P* < 0.001). For XBB, no demographic or clinical variables were associated with infection. In contrast, samples collected in 2023 had higher odds of XBB infection compared with 2022 (β = 3.921, *P* < 0.001), and residence outside Beirut was associated with increased odds (β = 1.004, *P* = 0.005). Several estimates, particularly for vaccination-related variables, were imprecise, reflecting sparse data and limited model stability.

**TABLE 4:** Relationship between predominant Omicron variants among all study participants and demographic and clinical characteristics^*a*^

Variant	Variables	Coefficient	Standard error	*P*-value^*b*^	95% Confidence interval
Omicron BA.5					
	Study participants (*n* = 356)				
	HCWs	Ref			
	Hospitalized patients	0.302	0.285	0.289	−0.256 to 0.861
	Gender (*n* = 297)				
	Male	Ref			
	Female	0.230	0.319	0.471	−0.395 to 0.855
	Age (*n* = 153)				
	≤60 years	Ref			
	>60 years	−0.330	0.393	0.401	−1.101 to 0.440
	Comorbid conditions (*n* = 139)	1.317	0.470	**0.005**	0.395 to 2.239
	SARS-CoV-2 vaccination status (*n* = 193)	−0.253	0.567	0.655	−1.365 to 0.858
	SARS-CoV-2 vaccine doses (*n* = 94)				
	≤2 doses	Ref			
	≥3 doses	0.056	0.540	0.917	−1.116 to 1.003
	Year of sample collection (*n* = 356)				
	2022	Ref			
	2023	−2.892	0.548	**0.000**	−3.968 to −1.817
	2024	−2.546	0.634	**0.000**	−3.789 to −1.303
	Site of sample collection (*n* = 356)				
	Beirut governorate	Ref			
	Other governorates	1.040	0.289	**0.000**	0.473 to 1.608
Omicron BA.2					
	Study participants (*n* = 356)				
	HCWs	Ref			
	Hospitalized patients	−16.967	705.006	0.981	−1,398.756 to 1,364.82
	Gender (*n* = 297)				
	Male	Ref			
	Female	0.207	0.297	0.471	−0.376 to 0.790
	Age (*n* = 153)				
	≤60 years	Ref			
	>60 years	−15.312	1,432.654	0.991	−2,823.261 to 2,792.637
	Comorbid conditions (*n* = 139)	Omitted			
	SARS-CoV-2 vaccination status (*n* = 193)	−16.192	783.430	0.984	−1,551.687 to 1,519.302
	SARS-CoV-2 vaccine doses (*n* = 94)				
	≤2 doses	Ref			
	≥3 doses	1.704	0.799	**0.033**	0.137−3.271
	Year of sample collection (*n* = 356)				
	2022	Ref			
	2023	−2.547	0.465	**0.000**	−3.460 to −1.634
	2024	−17.364	925.046	0.985	−1,830.422 to 1,795.693
	Site of sample collection (*n* = 356)				
	Beirut governorate	Ref			
	Other governorates	−1.438	0.340	**0.000**	−2.105 to −0.772
Omicron XBB					
	Study participants				
	HCWs	Ref			
	Hospitalized patients	17.237	583.564	0.976	−1,126.528 to 1,161.003
	Gender (*n* = 297)				
	Male	Ref			
	Female	−0.014	0.357	0.969	−0.714 to 0.686
	Age (*n* = 153)				
	≤60 years	Ref			
	>60 years	0.090	0.413	0.826	−0.719 to 0.901
	Comorbid conditions (*n* = 139)	−0.05	0.527	0.916	−1.088 to 0.977
	SARS-CoV-2 vaccination status (*n* = 193)	−16.192	4,020.251	0.997	−7,895.74 to 7,863.354
	SARS-CoV-2 vaccine doses (*n* = 94)				
	≤2 doses	Ref			
	≥3 doses	14.919	1,209.228	0.990	−2,355.124 to 2,384.964
	Year of sample collection (*n* = 356)				
	2022	Ref			
	2023	3.921	1.029	**0.000**	1.903−5.939
	2024	−13.603	1,308.214	0.992	−2,577.655 to 2,550.447
	Site of sample collection (*n* = 356)				
	Beirut governorate	Ref			
	Other governorates	1.004	0.360	**0.005**	0.297−1.712
Other Omicron variants	(Base outcome)				

^*a*^ Univariate logistic regression.

^*b*^ Bold values indicate statistically significant relationships (*P* < 0.05).

## DISCUSSION

This study builds upon our initial genomic surveillance efforts, which began in December 2021, and provides expanded insight into the molecular epidemiology of SARS-CoV-2 among HCWs and hospitalized patients in Lebanon (49). By extending our surveillance period to cover January 2022 through September 2024, we captured successive waves of variant emergence and replacement in both populations, offering a unique perspective into the evolutionary dynamics of SARS-CoV-2 across different clinical and occupational settings. Additionally, we highlighted the continued occurrence of breakthrough infections among hospitalized patients—and more notably among healthcare workers, who were among the earliest groups to be vaccinated.

The SARS-CoV-2 variants detected in Lebanon largely mirror global and regional evolutionary patterns, as reported by global genomic surveillance platforms such as Nextstrain (55) and GISAID (56). We observed a phased emergence and dominance of major Omicron sublineages over time, with BA.2-like variants predominating in early to mid-2022, followed by BA.5- and BA.2.75-like variants in late 2022, XBB-derived lineages (e.g., XBB.1.5, XBB.1.9, and XBB.1.16) throughout 2023, and increasingly divergent JN.1- and KP-derived lineages emerging in 2024. This temporal succession reflects sustained viral diversification and supports the role of sampling date in predicting variant circulation (57, 58).

While broad clade-level trends were consistent with global and regional data, lineage-level comparisons showed subtle differences. Several globally or regionally reported sublineages—such as BA.2.12.1, CH.1.1, EG.5.1, and HK.3—were not detected among our study groups, whereas others, such as EG.5.1.4 and LB.1.4, circulated in Lebanon while not prominently represented in global data sets. These distinctions may reflect region-specific introductions or local transmission dynamics, potentially driven by founder effects, stochastic spread, or variant-specific mutations acquired during community-level circulation (59–61). Our findings are also consistent with WHO reports on the phased emergence of Omicron sublineages, where BA.4 and BA.5 surged in late 2022, followed by diversification into XBB-related recombinants throughout 2023 and early 2024 (57, 62).

These findings suggest that the observed associations between variant distribution and factors such as sampling year and geographic location are more likely to reflect temporal changes in circulating lineages rather than intrinsic host susceptibility. The consistent association with sampling year across both populations supports this pattern, as successive Omicron sublineages emerged and replaced earlier variants during the study period. In healthcare workers, the geographic differences observed may reflect variation in the distribution of circulating variants across regions, rather than differences in individual risk factors. In contrast, the lack of consistent associations with demographic and clinical variables, particularly among hospitalized patients, suggests that variant distribution was not strongly determined by host characteristics in this cohort. However, these observations should be interpreted with caution, given the presence of unstable estimates for several variables, likely reflecting limited sample size and missing data.

The Centers for Disease Control and Prevention defines COVID-19 “breakthrough infections” as infections occurring 2 weeks following full vaccination (63). Our data revealed a significantly negative association between circulating SARS-CoV-2 variants and the number of administered vaccine doses among hospitalized patients, highlighting the importance of vaccine coverage and timely booster administration in reducing viral circulation and immune escape. However, a significantly positive correlation was detected among HCWs, supporting the role of waning immunity in facilitating breakthrough infections among high-risk groups. Our results advance the relationship between the evolution/emergence of new variants and vaccination. However, we have to cautiously interpret these data, especially with the complex interaction between immune escape and vaccination, whereby stochastic computational models suggest the inability of multiple vaccinations alone to prevent immune evasion and emergence of new variants (60).

Our cohort did not receive the bivalent mRNA COVID-19 vaccine (targeting ancestral and Omicron BA.4/BA.5 strains), and all breakthrough infections occurred at an average of 9.5 months and 12.5 months post-vaccination in 2022 and 2023, respectively—reflecting waning immunity over time. While none of the participants received updated boosters, earlier reports emphasized their clinical relevance with approximately 56%–57% effectiveness against COVID-19-related emergency department visits and hospitalizations during the period of BA.5 predominance (64). Additionally, the predominant detection of Omicron and its subvariants in our study aligns with broader observations within the MENA region. For instance, a multicenter study in Saudi Arabia reported a 2.73% incidence of breakthrough infections among fully vaccinated healthcare workers (65, 66). Similarly, in Qatar, Egypt, and the United Arab Emirates, despite high vaccination coverage, breakthrough infections were observed, particularly during waves of Omicron subvariant circulation, underscoring the challenges posed by immune-evasive variants (67, 68). Emerging evidence suggests that immune imprinting—where prior exposure to ancestral vaccine strains may blunt responses to newer variants—may contribute to reduced protection, particularly against XBB and JN.1 lineages (69).

Recent studies have demonstrated that emerging variants such as JN.1 and KP.3 exhibit enhanced immune evasion and altered receptor-binding properties. For instance, Cao et al. showed that JN.1 can evade key classes of neutralizing antibodies, including those effective against its descendant KP.3. Notably, both JN.1 and KP.3.1.1 were detected in our 2024 hospitalized cohort, reflecting their expanding global footprint (69). Furthermore, preclinical evaluations indicate that monovalent XBB.1.5-adapted boosters induce broader neutralization—particularly against XBB-derived lineages such as EG.5.1 and KP.3 families—than bivalent wild-type/BA.4-5 boosters (70). These findings reinforce the need to align vaccine strategies with evolving variant landscapes.

Taken together, these findings confirm the need for ongoing genomic surveillance in both clinical and occupational contexts. As new sublineages with established immune-escape potential continue to emerge as of the date of writing this manuscript, timely and geographically comprehensive data are critical to guide vaccine strategy and public health responses. Notably, NB.1.8.1 (“Nimbus”) was designated a variant under monitoring by the WHO TAG-VE in May 2025, exhibiting mutations (e.g., T22N, A435S, F456L, and T478I) associated with modest increases in immune evasion and ACE2 binding, though laboratory data indicate vaccine protection against severe outcomes remains effective (71). Furthermore, the recombinant variant XFG—designated a Variant Under Monitoring by the WHO in June 2025—has become dominant in parts of India and Southeast Asia, with a rising global share. While it harbors Spike mutations (e.g., F456L, T478I, N487D, and Q493E) associated with immune escape, current evidence suggests that it does not lead to increased severity, and vaccines remain effective against severe disease (72). These observations reinforce SARS-CoV-2’s capacity for antigenic drift and regional adaptation and underscore the critical importance of aligning vaccination strategies with the evolving variant landscape (73).

### Limitations

While our findings support the significance of genomic data and its contribution to regional data during a key period of SARS-CoV-2 evolution, this study has several limitations. The decline in PCR testing and increased reliance on rapid diagnostic tests may have led to underrepresentation of asymptomatic or mildly symptomatic cases, particularly in community settings. While HCWs represent a critical sentinel group, their early access to testing and vaccination may not fully reflect broader population trends. Additionally, clinical metadata for hospitalized patients was limited, precluding detailed correlation with variant type. Uneven sampling across time—especially in late 2023 and early 2024—may have also affected variant detection patterns. Finally, the use of two sequencing platforms (Illumina and Oxford Nanopore) may have introduced platform-specific variability despite quality control measures.

### Conclusion

Our study highlights the dynamic circulation of SARS-CoV-2 variants in Lebanon, aligning with global trends and emphasizing the importance of sustained genomic surveillance and data pathogen sharing. Our findings underscore the continued risk of breakthrough infections—particularly in the context of waning immunity and delayed booster uptake—and the emergence of immune-evasive variants. Despite limitations in surveillance and sequencing capacity, this study emphasizes the critical role of real-time genomic sequencing in guiding public health interventions and preparedness efforts.

## Data Availability

The data sets used and/or analyzed during the current study have been deposited in GISAID. The corresponding GISAID accession numbers for all sequences (hospitalized patients and healthcare workers) are publicly available on Zenodo at https://doi.org/10.5281/zenodo.20745983.

## SUPPLEMENTAL MATERIAL

Supplemental table and figures spectrum.02254-25-s0001.docx Table S1 and Figures S1 to S5.

ASM does not own the copyrights to Supplemental Material that may be linked to, or accessed through, an article. The authors have granted ASM a non-exclusive, world-wide license to publish the Supplemental Material files. Please contact the corresponding author directly for reuse.
