# Supplementary material for: Tracking the shifting landscape of SARS-CoV-2 variants in Lebanon among healthcare workers and hospitalized patients
Source: Microbiol Spectr. 2026 Jul 2;14(8):e02254-25. doi: 10.1128/spectrum.02254-25 (PMC13436191; doi:10.1128/spectrum.02254-25)
Supplement: Supplementary file 1 [file spectrum.02254-25-s0001.docx]

| **Variant** | **HCW 2022** | **HCW 2023** | **Hosp 2022** | **Hosp 2023** | **Hosp 2024** | **Global 2022–2024** | **MENA Region 2022-2024** |
| --- | --- | --- | --- | --- | --- | --- | --- |
| BA.1 | ✓ |  |  |  |  | ✓ | ✓ |
| BA.2 | ✓ | ✓ | ✓ |  |  | ✓ | ✓ |
| BA.4 | ✓ |  | ✓ |  |  | ✓ |  |
| BA.5 | ✓ | ✓ | ✓ |  |  | ✓ | ✓ |
| XBB |  | ✓ |  | ✓ |  | ✓ | ✓ |
| JN.1 |  |  |  |  | ✓ | ✓ | ✓ |
| JN.1-descendant |  |  |  |  | ✓ | ✓ | ✓ |

**Supplementary Table 1.** Distribution of SARS-CoV-2 Variant Categories across Study Groups along with global and Regional Trends

**Supplementary Figure 1.** Proportional distribution of detected SARS-CoV-2 lineages by month among HCWs (2022)**.**

**Supplementary Figure 2.** Proportional distribution of detected SARS-CoV-2 lineages by month among HCWs (2023)**.**

**Supplementary Figure 3.** Proportional distribution of detected SARS-CoV-2 lineages by month among Hospitalized patients (2022)**.**

**Supplementary Figure 4.** Proportional distribution of detected SARS-CoV-2 lineages by month among Hospitalized patients (2023)**.**

**Supplementary Figure 5.** Proportional distribution of detected SARS-CoV-2 lineages by month among Hospitalized patients (2024)**.**
